# Efficacy of stretching exercises versus transcranial direct current stimulation (tDCS) on task performance, kinematic and electroencephalography (EEG) spectrum in subjects with slump posture: a study protocol

**DOI:** 10.1186/s13063-023-07359-0

**Published:** 2023-05-24

**Authors:** Zahra Abdollahzade, Mohammad Reza Hadian, Roya Khanmohammadi, Saeed Talebian

**Affiliations:** Department of Physiotherapy, School of Rehabilitation, Tehran University of Medical Sciences, Tehran, Iran

**Keywords:** Slump posture, Mental fatigue, Electroencephalography, Transcranial direct current stimulation, Stretching exercise

## Abstract

**Background:**

Slump sitting is a common posture in workplaces. There is limited evidence that poor posture impacts the mental state. This study aims to investigate whether slump posture results in more mental fatigue during computer typing, compared with normal posture and also to compare the effectiveness of stretching exercises with tDCS in fatigue monitoring.

**Methods:**

The sample size for this study is set at 36 participants with slump posture and 36 participants with normal posture. In the first step, to find out the differences between normal and poor posture, they will be asked to perform the typewriting task for 60 min. During the first and last 3 min of typing, mental fatigue as the primary outcome using EEG signals and further measures including kinematic behavior of neck, visual analog fatigue scale, and musculoskeletal discomfort will be assessed. Post-experiment task performance will be calculated based on typing speed and typing errors. In the next step, to compare the effect of tDCS and stretching exercises on the outcome measures, the slump posture group will receive these interventions in two separate sessions before the typing task.

**Discussion:**

With the assumption of showing significant differences in terms of outcome measures between slump and normal posture groups and also by showing the possible changes of the measures, by using either tDCS as a central modality or stretching exercises as a peripheral modality; the findings may provide evidence to indicate that poor posture has adverse effect on mental state and to introduce the effective method to overcome mental fatigue and promote work productivity.

**Trial registration:**

Registered on the Iranian Registry of Clinical Trials on 21 September 2022, IRCT Identifier: IRCT20161026030516N2.

## Administrative information

Note: the numbers in curly brackets in this protocol refer to SPIRIT checklist item numbers. The order of the items has been modified to group similar items (see http://www.equator-network.org/reporting-guidelines/spirit-2013-statement-defining-standard-protocol-items-for-clinical-trials/).

**Table:** 

Title {1}	Efficacy of stretching exercises versus transcranial direct current stimulation (tDCS) on task performance, kinematic and electroencephalography (EEG) spectrum in subjects with slump posture: a study protocol
Trial registration {2a and 2b}.	Registered on the Iranian Registry of Clinical Trials on 21 September 2022, IRCT Identifier: IRCT20161026030516N2
Protocol version {3}	version is 1.0, Nov 2022
Funding {4}	The authors received no specific grant from any funding organization in the public, commercial or not-for-profit sectors for conducting this study.
Author details {5a}	Department of Physiotherapy, School of Rehabilitation, Tehran University of Medical Sciences, Tehran, Iran Zabdollahzade1990@gmail.com *Correspondence: hadianrs@sina.tums.ac.ir rkhanmohammadi@sina.tums.ac.ir talebian@tums.ac.ir
Name and contact information for the trial sponsor {5b}	There is no sponsor for the study.
Role of sponsor {5c}	Not Applicable, as there is no sponsor or funder

## Introduction

### Background and rationale {6a}

The employees are used to spending long periods of time in front of a computer with a dorsiflexed posture [1], in fact requiring high concentration and focus while working with a computer causes them not to pay attention to the body position. This lack of awareness of the body posture is one of the important reasons for poor posture among office workers [2].

Poor posture can change physiological, structural, physical, and functional aspects; furthermore, it can affect learning and attention [3]. For instance, slump posture decreases the rate of speaking compared to sitting upright [4] and slouched posture makes it difficult to do a math test [5]. According to Lee et al.’s study, forward head posture negatively influences the state of rest and concentration of the brain, as measured by a neuro-feedback system [6]. Based on these studies, it can be expected that poor posture causes or aggravates mental fatigue.

Mental fatigue is defined by feeling sleepy and drowsy while needing to concentrate [7] and has indicators such as feeling tired, a decrease in energy [8], mood changes [9], a decrease in performance, a decrease in accuracy and an increase in reaction time [9, 10], as well as changes in brain activity [7]. Mental fatigue has a negative impact not only on task performance in different environments, such as school [11] and workplace [12], but also on the safety of people [13]. For example; the risk of fatigue-related accidents increases, when employees get stuck in traffic after a long work day [14]. In some cases, such as those of industry workers, surgeons, drivers, and pilots, mental fatigue can lead to many hazards that cause both economic and human losses [12, 15]. Considering the high prevalence of mental fatigue in everyday modern life, it is necessary to search for efficient interventions to improve task productivity and occupational health. To date, the number of studies on tackling mental fatigue is fairly limited [16].

Exercise and physical activity have been considered as interventions for improving mental fatigue. These interventions increase the performance of the prefrontal cortex, which includes attention, memory, problem-solving, and decision-making [17]. For example, performing targeted stretching micro-breaks in surgeons without increasing the surgery time, improved their performance and concentration [18]. Furthermore, a short-term supervised exercise program including stretching exercises and joint mobility during a work shift has been shown to be more efficient than rest for reducing mental fatigue [19].tDCS is a type of non-invasive electrical stimulation that can change neural excitability in the cortex [20, 21]. tDCS can improve cognitive function in both healthy individuals and neurological patients [22, 23]. According to studies, anodal stimulation enhances learning, memory, and attention [24]. The effect of tDCS has been compared with caffeine as the most common habit to reduce fatigue. Interestingly, tDCS has a greater effect on attention and this effect also lasts longer (6 h) than caffeine (2 h) [25].

A limited number of studies have investigated the non-biomechanical part of poor posture. Most of these articles have used functional tests (such as rate of speaking) to investigate the impact of poor posture, and only one article has examined the correlation between forward head posture and brain signals as measured by a neuro-feedback system [6]; At present, there is an absence of studies utilizing QEEG to track mental fatigue in slump sitting, and in addition, the potential role of slump posture on work productivity needs to be investigated. Given the negative mental state consequences associated with poor posture and the high prevalence of slump sitting, there is a critical need to investigate changes in cortical activity and task productivity in this posture. Previous studies indicate that tDCS and stretching exercises can reduce the adverse effect of mental fatigue, however; no study has compared the effect of transcortical stimulation and stretching exercises in improving mental fatigue with neurophysiological markers of electroencephalography.

In this study, we will be trying to take a more comprehensive look at posture and assess its central aspect using brain data obtained from electroencephalography. Also, another aim of the study will be to investigate and compare the effects of transcortical stimulation and stretching exercises to reduce central complications caused by poor posture, determine the preferred intervention and introduce it for workplaces.

### Objectives {7}

The main objective of this study is to compare the mental fatigue through EEG during a prolonged typing task (i.e., 60 min) between subjects with slump and normal postures and to determine whether using tDCS or stretching exercises in the slump posture group, can deal with mental fatigue and minimize the difference in terms of mental fatigue between slump and normal posture groups.

Secondary objectives of the study include the following:

- To compare the kinematic parameters (i.e., entropy of neck motion and cervical angle), task performance (i.e., typing speed and errors), musculoskeletal discomfort, and visual analog fatigue scale between subjects with slump and normal postures.
- To compare the kinematic parameters (i.e., entropy of neck motion and cervical angle), task performance (i.e., typing speed and errors), musculoskeletal discomfort, and visual analog fatigue scale between slump posture following performing stretching exercise and normal posture.
- To compare the kinematic parameters (i.e., entropy of neck motion and cervical angle), task performance (i.e., typing speed and errors), musculoskeletal discomfort, and visual analog fatigue scale between slump posture following tDCS and normal posture
- To compare the kinematic parameters (i.e., entropy of neck motion and cervical angle), task performance (i.e., typing speed and errors), musculoskeletal discomfort, and visual analog fatigue scale between 3 conditions of stretching exercise, tDCS, and no intervention in slump posture group.

### Trial design {8}

**Fig. 1:**
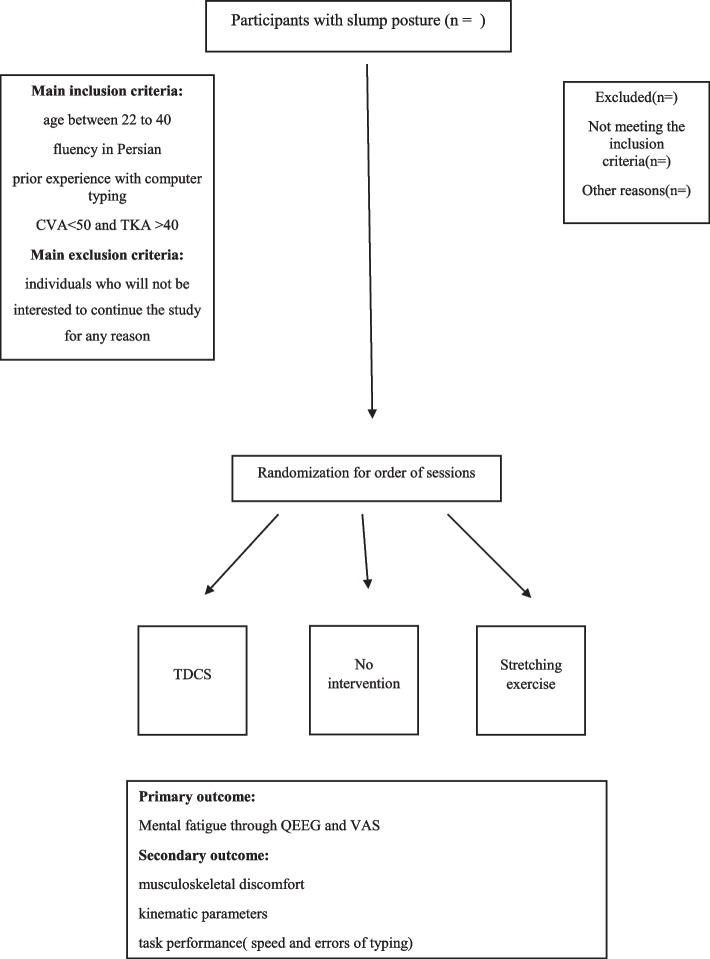
Flow chart of slump posture (CVA, craniovertebral angle; TKA, thoracic kyphosis angle; tDCS, transcranial direct current stimulation; QEEG, quantitative electroencephalography; VAS, visual analog scale)

This is a single-center, assessor-blinded study with two parts; the first part is exploratory research with 1:1 allocation, designed to compare the outcome measures between the normal and slump posture individuals, and the second part is a crossover randomized controlled trial with a superiority design which aims to compare the effects of tDCS and stretching exercises to improve the outcome measures of the study. The study will be performed between October of 2022 and March of 2023. All the outcome measurements will be assessed during the first (T1) and last (T2) 3 min of typing. Figure 1 shows the slump posture group flowchart.

## Methods: participants, interventions, and outcomes

### Study setting {9}

The sample size is set at 36 subjects with slump posture and 36 subjects with normal posture. Participants will be recruited from TUMS (Tehran University of Medical Sciences). The present study will be conducted in the motor control laboratory at the School of Rehabilitation of TUMS. Tehran, Iran. Participants will be familiarized with the study, and they will practice with the computer. After a short break, the experiment procedures will begin.

#### Task

Previous research showed that working with a computer for a period of 45 to 60 min had reduced the performance to its minimum level [26]. So, in this study, 60 min are considered for typing, and spell-check of the word program will be turned off for all conditions. Three different Persian texts from a single book are selected for the study. The same text will be used for individuals with normal and slump posture (i.e., no intervention). But for the intervention sessions, the text will be changed to prevent the learning effect.

### Eligibility criteria {10}

Subjects will be included, if they meet the following criteria: age between 22 and 40 years, have a BSc degree, fluency in Persian, prior experience with computer typing and lack of 10-finger typing skills, no history of shoulder and spine surgery or fracture, no obvious scoliosis, body mass index (BMI) < 30. Postural angles including the craniovertebral angle (CVA) and the thoracic kyphosis angle (TKA) are considered as follows: CVA ≥ 50 and TKA between 20 and 40 for the normal posture group, CVA < 50 and TKA > 40 for the slump posture group. In addition, cardiac or metabolic diseases, severe head injury, metals in the head, psychological problems, seizures, a history of taking medications that affect the central nervous system, and pregnant women will not be included in the study.

The typewriting background will be controlled by a self-report of daily amount of computer usage and years of computer use. There will be no significant difference between the two groups with respect to computer usage.

Before the tests, subjects are asked to follow these: (1) proper and adequate sleep, (2) avoid using drugs or alcohol in the last prior week, and coffee in the last prior 8 h.

Excluding criteria: those individuals who will not be interested in continuing the study for any reason.

#### Postural angle measurements

Photogrammetry is a safe, accessible, and inexpensive technique, that provides an accurate postural angle measurement [27]. Postural angles as a baseline criterion will be measured. For this, the spinous processes of the seventh cervical (C7) and twelfth thoracic (T12) vertebrae and tragus will be marked by palpation, and reflexive markers will be attached to these points. The camera will be placed 120 cm away from the participant at a height of 130 cm [27]. The photography will be done from the sagittal plane with a camera (SAMSUNG, A50, South Korea). The photograph will then be imported to AutoCAD software version 24. To assess the posture of the head, we will use CVA, which is obtained by the intersection of a horizontal line passing through the C7 marker and a line joining the tragus to the C7 marker [28]. To measure the angle of thoracic kyphosis, we will draw a straight line between C7 and T12 markers, and then a perpendicular line is drawn on each of the lines. The intersection of the perpendicular lines makes an angle which is considered TKA [27].

### Who will take informed consent? {26a}

At the start of the study, the investigator will evaluate the subjects for inclusion and exclusion criteria, explain the study in detail and answer the questions. Then all participants will be asked to read and sign the consent form which is approved by the Ethics Committee of TUMS with the ID IR.TUMS.FNM.REC.1401.069. This form has information such as the title and goals of the study, the names of researchers, research background, how the interventions will be conducted, and obligations.

### Additional consent provisions for collection and use of participant data and biological specimens {26b}

The study will not involve collecting biological specimens therefore, there is no need for additional consent provisions. All the steps of this research will be done based on the consent form which explains what study participation entails, the research process and the potential benefits and risks of the study.

## Interventions

### Explanation for the choice of comparators {6b}

The normal posture group will participate just once while the slump posture group will carry out the experiment in 3 separate sessions (i.e., no intervention, stretching exercise, and tDCS) with a week interval between sessions. The sequence of sessions will be randomized. The assessor involved in analyses will be blind to groups and conditions.

### Intervention description {11a}

Stretching exercises and tDCS are performed by one expert and trained physiotherapist with 8 years of experience in these fields. The interventions take a total of 10 min, and individuals in the slump posture group will receive exercises or tDCS, before typing in one of the three sessions of the study.

#### Stretching exercise

Exercise is a recommended intervention for computer users [29]. It was reported that stretching exercises had become more accepted in workplaces [30]. Therefore, three sets of nine stretching exercises will be used to improve the flexibility of muscles in the neck, shoulder, upper limbs, and back. These exercises are selected based on the review of Lee et.al, who assessed different types of exercises for VDT (Video Display Terminal) operators and office workers [31]. It takes a total of 10 min to perform the exercises, and individuals in the slump posture group will do these exercises before typing in one of the three sessions of the study. They are taught to stretch the muscles slowly and constantly until they feel mild discomfort, and then hold this position for 15 s. A break of 60 s is considered between sets.

#### tDCS

To provide transcranial direct current stimulation, an Oasis Pro DC stimulator (Mind Alive company, Canada) will be used. According to previous studies, stimulation of the left dorsolateral prefrontal cortex (DLPFC) makes improvements in cognitive functions such as decision-making, attention, and working memory [32–38]. Therefore, the anode is placed over F3 according to the 10–20 system, and the cathode is placed on the supraorbital region of the opposite side. A current of 2 mA is applied through two 3 cm × 5 cm conductive rubber electrodes inside a sponge soaked with physiologic saline [39]. The stimulation increases during a 30 s ramp up to 2 mA, it is stabilized for 10 min, then reduces to zero over the last 30 s. At the time of receiving current by participants, continuous monitoring of the output current will be done.

### Criteria for discontinuing or modifying allocated interventions {11b}

If subjects report seven or higher level of itching, tingling, and heat based on 10-point Likert scale, tDCS will be stopped.

### Strategies to improve adherence to interventions {11c}

Stretching exercises and tDCS will be performed in two separate sessions under the supervision of an experienced physiotherapist.

### Relevant concomitant care permitted or prohibited during the trial {11d}

The subjects will be told that all relevant concomitant care and treatments such as postural corrective exercises, are prohibited during participation in this study**.**

### Provisions for post-trial care {30}

No mental or physical damage is anticipated due to evaluations or interventions. However, if people are harmed during this study because of evaluations or interventions, this will be reported to the Tehran University of Medical Sciences ethics committee and the research team of this study will take responsibility for free treatment.

### Outcomes {12}

#### Task performance

Task performance will be obtained by typing speed (i.e., characters per minute) and typing errors (i.e., errors per minute), which will be calculated by the assessor at the end of the tests.

#### Subjective assessment

Musculoskeletal discomfort will be measured through a visual analog discomfort scale and a body-part diagram divided into 15 regions (i.e., neck, upper back, lower back, left and right sides of the shoulder, forearm, wrist, thigh, knee, and lower leg). Participants will be asked to mark their musculoskeletal discomfort based on a 10-cm horizontal line (from 0 to 10 cm which indicates “no discomfort” and “extreme discomfort,” respectively) and mark the location of pain on the body-part diagram [40].

Mental fatigue will be measured using a visual analog fatigue scale. This scale is a horizontal line ranging from 0 to 10 cm. Participants will be asked to mark the line based on the intensity of their mental fatigue (from 0 cm showing ‘no mental fatigue’ to 10 cm indicating ‘maximal mental fatigue’) [41].

#### Kinematic parameters

Kinematic properties including the non-linear dynamical features of neck motion and cervical angle changes will be measured. For this, markers will be placed on the tragus, spinous process of C7, and corner of the eye. A SAMSUNG (A50, South Korea) camera (30 frames per second) will be placed at a distance of 2.18 m from the participant and a height of 1.25 m [42]. In the first and last three minutes of typing, a recording will be taken. Then the recording will be imported to Kinovea software version 0.8.15.

Mean and standard deviations of CVAs will be used to assess the cervical angle changes, and Sample Entropy will be calculated for neck motion Entropy. Entropy Analysis will be calculated in Excel software using SEN = -ΣPi ln Pi formula for each marker, where Pi is the spectral amplitudes of path bin i, Σ Pi = 1, and ln = denotes the natural logarithm.

#### Electroencephalography

The brain activities will be recorded using a 64-channel EEG system (EB Neuro, Italy) with a sampling rate of 1024 Hz and bandwidth of 0.2–70 Hz. The Ag/Ag Cl surface electrodes with a diameter of 8 mm and an impedance of less than 20 kHz, will be distributed throughout the whole head of the participants. The 21 electrodes, according to the international 10/20 system, will be placed in Fp1, Fpz, Fp2, F7, F3, Fz, F4, F8, T3, C3, Cz, C4, T4, T5, P3, Pz, P4, T6, O1, Oz, O2. A reference and ground electrode will be placed on the mastoid process and the wrist of the right hand respectively. To record vertical and horizontal eye movement, a pair of electrodes will be placed above and below the right eye and a pair of electrodes will be placed at the corner of each eye. Blinking and eye movement artifacts will be monitored with the electrooculogram and the O1 and O2 electrodes. The EEG will be recorded for 3 min under two conditions: while subjects will rest with their eyes open and while they will be typing, during the first and last 3 min of continuous typing. Thereafter, raw EEG data will be imported into LORETA version 2015–12-22 to extract the frequency domains (i.e., Fast Fourier transform, FFT) for delta, theta, alpha, beta, and gamma rhythms, by 1024 sampling frequency and two epochs (2048) for 21 electrodes. Then the relative power levels of each band will be calculated.

### Participant timeline {13}

**Fig. 2:**
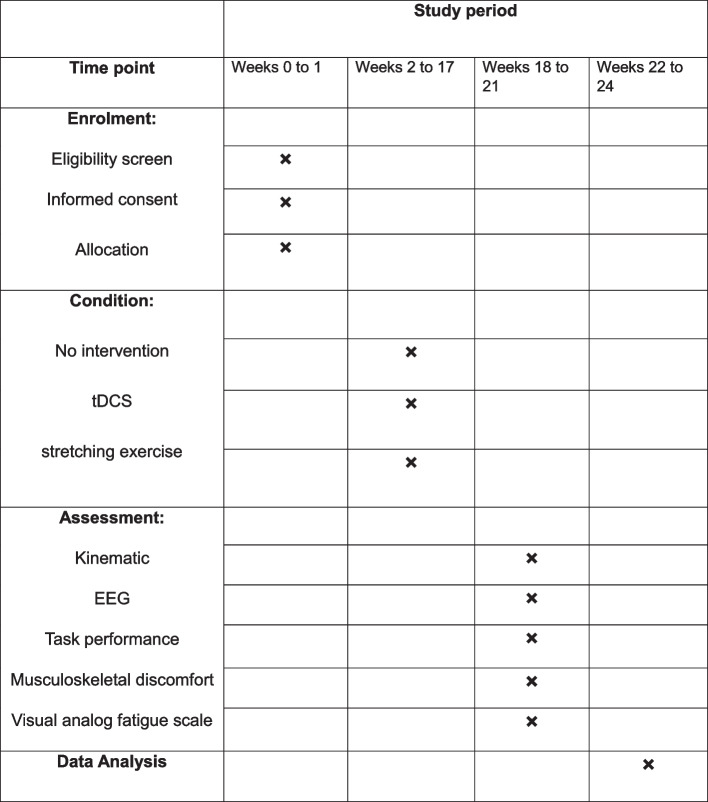
The schedule of study enrollment, interventions, and assessments (according to SPIRIT)

The study will be performed between October of 2022 and March of 2023. The details of the schedule of enrollment, interventions, and assessments (according to the SPIRIT) are shown in Fig. 2.

### Sample size {14}

Based on pilot data of 12 subjects (i.e., 6 cases for slump posture and 6 cases for normal posture), the sample size was calculated using G*Power 3.1.9.4 (Düsseldorf, Germany) software. Sample size estimation was conducted according to one-tailed testing with an alpha set at 0.05 and the desired power of 0.90. For the first part of the study, a sample size of 50 participants distributed in two groups was estimated. For the second part, we calculated that 30 subjects with slump posture, are needed.

To consider a potential 20% drop-out rate, the sample size is 72 participants (36 in each group).

### Recruitment {15}

Participants including students and employees of TUMS, will be recruited through online advertisements on the social media and advertising posters on bulletin boards of schools and dormitories of TUMS. Volunteers to participate in the study will be referred to the motor control laboratory at the School of Rehabilitation of TUMS. These people will be checked in terms of inclusion and exclusion criteria and a final decision will be made.

## Assignment of interventions: allocation

### Sequence generation {16a}

Randomization sequence will be generated using a web-based randomization service (https://statpages.info/latinsq.html). Given three states for slump posture group (i.e., no intervention, tDCS, and stretching exercise), there will be six different possible orders (ABC, ACB, BAC, BCA, CAB, and CBA). Each order will be performed five times in an equal ratio.

Randomization will be done at TUMS. Access to the randomization schedule will be restricted to an investigator who is not involved in the recruiting process.

### Concealment mechanism {16b}

The random allocation sequence is printed onto papers which will be then sealed in opaque envelopes.

### Implementation {16c}

Enrolment including eligibility screening, informed consent, and allocation, interventions will be conducted by a physiotherapist who is not involved in the analysis of data.

## Assignment of interventions: blinding

### Who will be blinded {17a}

This is a single-blind study. The assessor who analyses the outcomes and performs the statistical analyses will be blind to groups and conditions and will not participate in taking measurements.

### Procedure for unblinding if needed{17b}

The study is primarily an open-label trial with only outcome assessors being blinded so there is no need for an unblinding procedure.

## Data collection and management

### Plans for assessment and collection of outcomes {18a}

Data collection will be performed by one of the investigators of study who will be trained in how to do assessments. Subjects will be familiarized with the equipment and tests of study during screening. For all participants, data collection will be conducted at a certain time before noon, in face-to-face meetings at the motor control laboratory at the School of Rehabilitation of TUMS.

Participants will receive a SMS text or an email to remind their appointment the day before. Principal investigator of the study will supervise data collection to check the quality, completeness, plausibility, and possible errors of the collected data.

### Plans to promote participant retention and complete follow-up {18b}

The commuting expenses of the participants will be paid and flash memories will be provided to thank them.

### Data management {19}

At the beginning of the study, participants will be assigned a unique identification code and the data sent to the study assessor will only include the code without participant identification information. Subjective assessments and demographic information collected on paper forms will be entered into specific Excel files and a second study staff member will check the data entry process for accuracy. All paper-based data for the study including consent forms, identifiable information, and questionnaires will be kept separate from each other in a locked cabinet in the motor control laboratory at the School of Rehabilitation of TUMS. The electronic records of the study, i.e., raw data of EEG, Entropy, and the Excel files stored on a password-protected external hard drive and computer. Both paper and digital documents will only be accessible to the research team.

### Confidentiality {27}

To protect the privacy of all participants, they will be allocated an individual trial identification code, the research data will be stored on a password-protected external hard drive and computer and only members of the study team will access to the data.

### Plans for collection, laboratory evaluation, and storage of biological specimens for genetic or molecular analysis in this trial/future {33}

See above 26b- there will be no biological specimens stored in this research study.

## Statistical methods

### Statistical methods for primary and secondary outcomes {20a}

Data analysis will be performed using SPSS, version 22 (IBM, NY, USA). Baseline characteristics such as age, weight, height, BMI, years of computer use, and daily computer usage will be summarized with standard descriptive statistics. Depended variables will be described with means and standard deviations. The normality of distribution will be checked by Kolmogorov–Smirnov.

Multivariate ANOVA (MANOVA) will be used to compare the variables between correct and slump posture groups.

Statistical analysis of the outcomes of 3 conditions in the slump posture group will be conducted using repeated measures ANOVA and LSD (least significant difference) post hoc test. The significance level of tests will be considered at 0.05. Mean difference and standardized mean difference and their 95% confidence intervals (Cohen’s D) will be calculated as effect sizes.

### Interim analyses {21b}

There will be no stopping guidelines and interim analyses, as this is a low-risk study with no anticipated problems for participants.

### Methods for additional analyses (e.g., subgroup analyses) {20b}

There will be no additional analyses for the present study.

### Methods in analysis to handle protocol non-adherence and any statistical methods to handle missing data {20c}

Outcome data obtained from protocol non-adherence will not be considered in the study. We will make all efforts to avoid missing data and conduct analysis on complete cases; however, if missing data is more than expected (more than 5%), an intention-to-treat analysis will be performed.

### Plans to give access to the full protocol, participant-level data, and statistical code{31c}

On completion of the study, the full protocol, the datasets analyzed during the current study, and the statistical code will be made available to other researchers upon reasonable collaborative request to the corresponding author.

## Oversight and monitoring

### Composition of the coordinating center and trial steering committee {5d}

The coordinating center and steering committee are composed of independent experts and study investigators. They will meet once every 2 months to oversee all aspects of the study and to monitor the safety and progress of the study. ZA will perform the participant recruitment and data collection for the study. All four authors are responsible for the study conduction and final results and reports. When problems occur, they will share information and MRH who is the principal investigator and in charge of supervising the study team, will make the final decisions.

### Composition of the data monitoring committee, its role and reporting structure {21a}

The data monitoring committee will not be formed due to the safety of the evaluation and intervention procedures of the study.

### Adverse event reporting and harms {22}

No mental or physical damage to the participants of this study is anticipated. However, if people are harmed during this study because of evaluations or interventions, this will be reported to the Tehran University of the Medical Sciences ethics committee and the research team of this study, will take responsibility for free treatment.

### Frequency and plans for auditing trial conduct {23}

The project management group will have regular audits to monitor trial-related activities and documents.

### Plans for communicating important protocol amendments to relevant parties (e.g. trial participants, ethical committees) {25}

Any change in the study plan requires the approval of the Ethical Committee.

### Dissemination plans {31a}

All the results obtained from the present research will be published in some articles.

## Discussion

To the best of our knowledge, this is the first study to investigate the impact of poor posture on mental fatigue.

Different techniques have been employed to measure mental fatigue. These methods are classified subjectively, behaviorally, and physiologically [43].

Subjectively, decreased energy [8], alertness [44], or motivation [45] with an increase in feelings of tiredness [8] are the characters of mental fatigue. In this method, standard questionnaires such as F-VAS have been used [43]. Behaviorally, performance deterioration including decreased in accuracy and slower responses, occurs following mental fatigue [9, 10, 46]. Effects of mental fatigue on performance have been studied since the beginning of the nineteenth century; Different theories about cognitive mechanisms have been proposed in this issue. According to these studies, people begin to experience mentally tired and their performance deteriorates when the costs of doing a task overtake the benefits of finishing that task [13]. Performance can be measured in different ways; For employees who are prone to mental fatigue, computer typing provides information about the level of mental fatigue without interfering with regular work activities [13, 47]. Physiologically, mental fatigue is detected by tracking alternations in the neural activity of the cortex [9, 48–50]. EEG spectral band change has been considered a reliable biomarker to detect mental fatigue. Increased slow-band EEG activity (i.e., 4–13 Hz), is associated with decreased mental activity [7].

Recent studies have revealed that mental fatigue has a negative effect on neuromuscular function including strength, muscle activity, and joint steadiness [51]. According to Marcora et al. (2009), during the condition of mental fatigue, people experience greater discomfort, decreased muscle endurance, and a higher perception of effort [46]. These changes in neuromuscular function may lead to loading of the musculoskeletal system and changes in kinematic parameters.

The hypothesis of altered cognitive processing in poor posture has been reported in previous studies although there is a small number of studies on this topic. For instance, better scores of verbal episodic memory was associated with more upright posture, which showed that the cognition of older adults was affected by their posture [52]. With these evidences, it is possible that typing task affects normal and slump posture individuals differently in terms of mental fatigue and kinematic parameters.

In this regard, by comparing tDCS and stretching exercises to monitor the adverse effects of poor posture, we are hopeful that results may contribute towards decision-making for workplaces and may be used as a basis for health recommendations of office workers.

## Trial status

The protocol version is 1.0 (September 21, 2022). Recruitment began on October 1, 2022. We expect that recruitment will be completed on February 5, 2023.

The datasets of study participants are available through the corresponding author upon reasonable request.

## Data Availability

The datasets of study participants are available through the corresponding author upon reasonable request.

## Abbreviations

tDCS: Transcranial direct current stimulation
QEEG: Quantitative electroencephalography
TUMS: Tehran University of Medical Science
VAS: Visual analog scale
CVA: Craniovertebral angle
TKA: Thoracic kyphosis angle
BMI: Body mass index
VDT: Video Display Terminal
FFT: Fast Fourier transform
LSD: Least significant difference
